# Enhancing Biophysical Muscle Fatigue Model in the Dynamic Context of Soccer

**DOI:** 10.3390/s24248128

**Published:** 2024-12-19

**Authors:** Arian Skoki, Stefan Ivić, Sandi Ljubic, Jonatan Lerga, Ivan Štajduhar

**Affiliations:** 1 Department of Computer Engineering, Faculty of Engineering, University of Rijeka, Vukovarska 58, 51000 Rijeka, Croatia; arian.skoki@riteh.uniri.hr (A.S.); sandi.ljubic@riteh.uniri.hr (S.L.); ivan.stajduhar@riteh.uniri.hr (I.Š.); 2 Department of Fluid Mechanics, Faculty of Engineering, University of Rijeka, Vukovarska 58, 51000 Rijeka, Croatia; stefan.ivic@riteh.uniri.hr; 3 Center for Artificial Intelligence and Cybersecurity, University of Rijeka, R. Matejcic 2, 51000 Rijeka, Croatia

**Keywords:** muscle fatigue, mathematical model, optimization, simulation and modeling, soccer

## Abstract

In the field of muscle fatigue models (MFMs), the prior research has demonstrated success in fitting data in specific contexts, but it falls short in addressing the diverse efforts and rapid changes in exertion typical of soccer matches. This study builds upon the existing model, aiming to enhance its applicability and robustness to dynamic demand shifts. The objective is to encapsulate the complexities of soccer dynamics with a streamlined set of parameters. Our refined model achieved a slight improvement in the $R^{2}$ score in the maximum hand-grip test, increasing from 0.87 to 0.89 compared to the existing model. It also demonstrated dynamic change robustness in a soccer-specific 1 min drill and 15 min treadmill protocol extracted from the literature. Through individualized fitting on a 10-repetition 80 m sprint test for a soccer player, the model exhibited $R^{2}$ scores between 0.62 and 0.80. Furthermore, when tested with actual soccer match data, it maintained a robust performance, with the average $R^{2}$ scores ranging from 0.70 to 0.72. The proposed approach holds the potential to advance the understanding of tactical decisions by correlating them with real-time physical performance, offering opportunities for more informed strategies and ultimately enhancing team performance.

## 1. Introduction

Soccer is a dynamic sport where the same starting team lineup can yield different results due to various factors, such as psychology, conditioning, injuries, and weather conditions [1]. Each athlete provides a unique set of attributes, encompassing running speed, acceleration, jumping ability, and overall game-play proficiency, which collectively determine their physical capabilities. During the preseason, coaches often employ various tests, such as repeated sprinting, the Yo-Yo test, the 30-15 intermittent test, and functional movement screens, to gather valuable information about the players [2,3]. These tests provide a foundation for further analysis but are not conclusive. However, they provide an excellent framework for identifying the optimal parameters for MFMs [4], enhancing the understanding of players’ exertion levels during matches, and explaining the effect of high-intensity actions on match outcomes [5].

MFMs are categorized into theoretical and empirical models. Theoretical MFMs are rooted in presumed physiological processes, while empirical models are derived from observations and experimental data [6]. Given soccer’s unpredictable nature, mathematical models should simplify information for a better understanding of the game.

Wearable devices with sensors such as GPS, accelerometers, and gyroscopes quantify physical effort in soccer [7,8]. These devices enable practitioners to evaluate athletes’ performance during training sessions and matches using aggregate metrics like total distance covered, sprinting distance, and the number of accelerations and decelerations. These metrics provide valuable feedback for planning session loads and optimizing players’ workloads.

Beyond aggregate metrics, sensors also provide continuous data with timestamps, enabling us to track a player’s speed at each moment during training and matches. These granular data are suitable for determining the parameters in MFMs. These models can assist practitioners in articulating athletes’ fatigue and recovery profiles and visualizing players’ expenditure throughout the match. Section 1.1 will discuss the current state of MFMs and their limitations in soccer.

### 1.1. Related Work

One of the most important MFMs was developed by Liu, Brown, and Yue [9] (LBY). This model used fatigue (*F*) and recovery (*R*) parameters to explain muscle activation, fatigue, and recovery during voluntary effort. To test the model, the participants performed a hand-grip as hard as possible for as long as they could. The results showed that the model could predict muscle force production, suggesting that, under maximum effort, only 97% of the true maximum force was achievable. This experiment demonstrated the model’s effectiveness in describing muscle behavior during voluntary effort, fatigue, and recovery.

LBY’s three-compartment model was further refined with resting, active, and fatigued compartments by Xia et al. [10]. This model effectively predicted fatigue and recovery for various activation patterns and offered a computationally efficient method. However, it has limitations, particularly in addressing dynamic strength surfaces and central fatigue representation, and should be used cautiously for prolonged or high-intensity tasks involving eccentric motion. Alternative efforts were undertaken to broaden the Xia et al. [10] approach by introducing varied dependencies that tailored it to specific tasks; however, these modifications proved to be primarily effective in the context of maximal efforts [11,12,13].

SNS [14] introduced a four-compartment model, overcoming the limitations of regression-based models. Governed by differential equations with key parameters representing the total force-generating capacity ($M_{0}$), fatigue rate (F), and recovery rate (R), the model offers a comprehensive understanding of activation physiology. Valid for various exercises, the model predicts muscle output accurately ($R^{2}$ ranging from 0.81 to 1.00). However, it lacks constraints on the speed of change for active motor units, potentially leading to unrealistic activation rates.

An extension of Frey-Law’s research investigated the impact of using half of the MVC on a muscle fatigue prediction model [15]. The model maintains accuracy when the MVC values are evenly distributed or split between the start and end. However, when relying exclusively on the first half of the MVC, the model introduces errors, emphasizing the need for proper data sampling.

Various MFM phenomena have been investigated, including different activities like maximum hand-grip contraction [9,14], push-type work commonly encountered at construction sites [16], quadriceps contraction [14,17,18], knee contraction [19], and sprinting and cycling [14]. Our goal is to extend the application of MFMs to the field of soccer. In summary, multiple MFMs exist, each with its set of challenges, including the following:

- Complex parameter selection and implementation;
- Suitability primarily for maximum effort or non-dynamic changes in effort;
- Limited data for result reproducibility and comparison.

### 1.2. Contributions and Structure

Among the models discussed in Section 1.1, the SNS approach stands out for its simplicity, applicability, and adaptability to soccer dynamics. However, it requires refinement to be effectively applied in this domain. Thus, we have opted to extend and refine this approach, ensuring its resilience to the varied demands of a soccer match. To the best of our knowledge, no such model has been specifically applied in the context of soccer. In alignment with the aforementioned goals, the primary contributions of this paper are as follows:

- Adjustment of the SNS approach to accommodate dynamic changes in $M_{AD}$ (representing the active units required by the brain based on the athlete’s goals during exertion);
- Optimizing the MFM parameters during the repeated sprinting test to show its efficacy in a soccer match;
- The provision of full access to the dataset utilized in this manuscript and the accompanying code.

This paper is organized as follows. Section 2 details the data acquisition and processing, outlining the model modifications and parameter optimization steps. Section 3 presents the outcomes and model performance across multiple optimization runs and matches. Subsequently, Section 4 provides a discussion of the results, and Section 5 concludes with some final remarks.

## 2. Materials and Methods

This section covers the data used for validating the proposed approach and evaluating the MFM. It explains the mathematical enhancements made to the model and the reasoning behind them. The section also details the parameter optimization methodology, explaining how optimal values were determined to accurately represent the player. Additionally, it provides an example workflow demonstrating how the model can be applied to gain insights into player performance. For transparency and accessibility, all the data and source code used for generating figures, tables, and running optimizations are publicly accessible at the following link: https://github.com/askoki/soccer-fatigue-model (accessed on 4 December 2024).

### 2.1. Study Design

Data collection originated from two distinct sources: scientific studies and a professional soccer club competing in a top-tier national division. Related work data encompassed results from a maximal hand-grip test by LBY, a soccer-specific 1 min drill [20], and a 15 min intermittent treadmill protocol [21]. This dataset facilitated model comparisons and the identification of limitations within existing research. Concurrently, data sourced from a professional soccer club offered insights into the model’s performance in a typical sporting environment. External load data were recorded using a GPexe pro^2^ wearable sensor (Exelio srl, Udine, Italy) operating at a sampling rate of 18 Hz. Firstly, a preseason 80 m sprint repeatability test was conducted to evaluate player performance, followed by the recording of four matches using the same wearable technology.

### 2.2. Data Acquisition

The data from all scientific studies were extracted using the Webplotdigitizer software, Version 4.6 [22], which enabled data extraction in a standardized format using comma-separated values (CSV). As discussed in Section 1.1, numerous MFMs have been subjected to testing with maximal effort trials, yielding satisfactory outcomes. Among the pioneers in the MFM approach, LBY conducted a seminal maximal hand-grip test, making it a crucial inclusion in our study. Additionally, data from two other studies were examined for their relevance to soccer applications. Firstly, data from a 1 min soccer-specific circuit performed by a representative player were extracted from the study by Savoia et al. [20]. This circuit included 4–6 s (21.2 m) sprints with CIDs every 5.6 m, 10 s (40 m) of linear striding, 10 s (28 m) of slalom runs, and 10 s (20 m + 20 m) shuttle runs. The final test data were extracted from the study by Greig and Siegler [21], which investigated soccer-specific fatigue and hamstring strength. The study provided data during a 15 min soccer-specific intermittent treadmill protocol. All three tests were utilized to evaluate the model’s stability during dynamic changes and to compare its performance with similar work by SNS.

The professional soccer club data involved preseason screening tests, where players engaged in maximum effort running over an 80 m distance, repeated 10 times. The test was conducted on a well-maintained grass surface under favorable weather conditions. The temperature during the test was 26 °C, with clear, sunny skies ensuring consistent visibility. A light breeze, averaging 5 km/h, provided mild airflow without significantly influencing the athletes’ performance. The maximum effort running data were gathered at the beginning of the 2023/2024 season, with ethical approval obtained through the Declaration of Helsinki from the University of Rijeka (REF: 2170-1-43-29-23-2). Five players, representing various playing positions such as center back, full back, midfielder, forward, and wing forward, willingly participated in the study.

Following the screening, the players progressed to the second stage, involving their participation in four friendly or official matches over the next two months. Throughout both stages, the players were equipped with FIFA-approved GPexe pro^2^ devices featuring a sampling frequency of 18 Hz. This was made possible by a lightweight vest that had a small device attached to the back, ensuring the players could wear it conveniently. The wearable sensors are outfitted with GPS, accelerometer, magnetometer, and gyroscope functionalities, facilitating the extraction of diverse metrics such as total distance covered, number of sprints, impact forces, maximal speed, and more.

However, such metrics are available only in a cumulative format, with the smallest interval being 5 min. To overcome this limitation, time course data were extracted from the sensors, providing data samples at an 18 Hz frequency. Each data point contained information about speed, timestamp, latitude, and longitude. Hence, speed served as the foundation for all calculations, further enhanced by player weight, a topic that will be extensively explored in Section 2.3.

### 2.3. Data Preprocessing

The raw data collected from GPexe pro^2^ devices had a sampling frequency of 18 Hz, resulting in a time series of players’ velocities recorded every 55 ms. To enhance data smoothness, we calculated the average speed over each one-second interval. In line with the model proposed by SNS [14], our extended model requires specific input values, particularly the velocities players aim to achieve.

**Figure 1:**
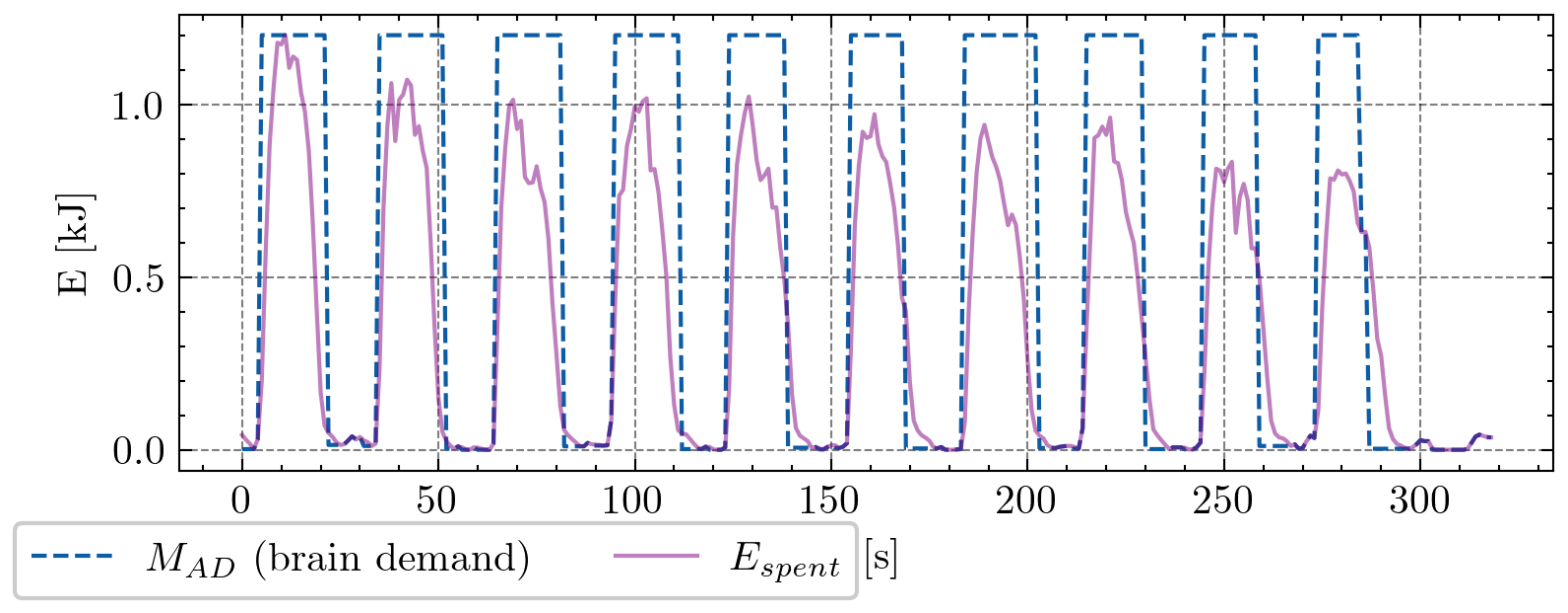
Visualization of the intended energy expenditure ($M_{AD}$) and spent energy ($E_{spent}$) during the 80 m sprint test. Blue dashed line represents the peak energy attained during the first sprint, considered as the desired energy for the subsequent sprints. X-axis denotes time in seconds, and y-axis indicates energy in kilojoules. Violet solid line shows kinetic energy calculated directly from the measured speed and athlete mass.

**Figure 2:**
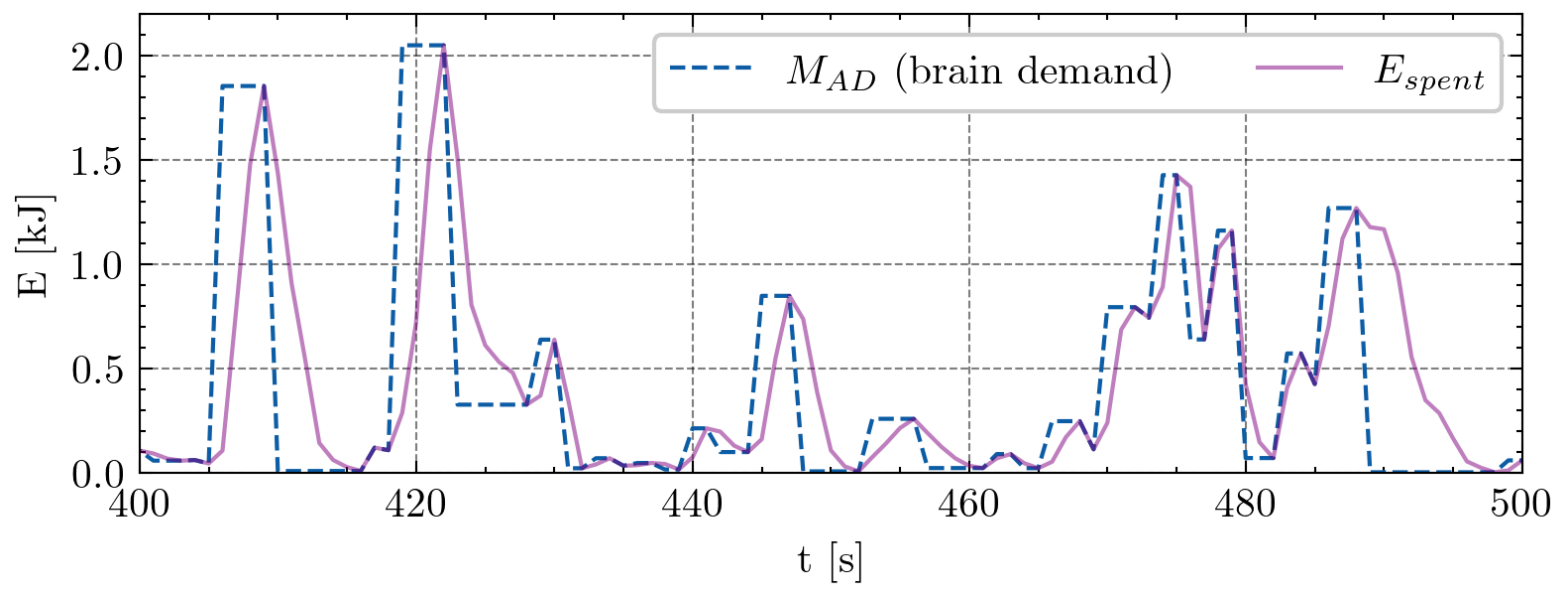
Visualization depicting the determination of desired energy $M_{AD}$ during a 100 s snippet from the match. Blue dashed line marks the assessed desired energy ($M_{AD}$), while violet solid line represents measurements from the sensors (kinetic energy, $E_{spent}$). X-axis values represent time in seconds, and y-axis values indicate energy in kilojoules. Two key assumptions guided this determination: (a) a player’s desired speed aligns with the maximum of the acceleration interval, and (b) the player’s intention to decelerate corresponds to the minimum slope value of decreasing velocities.

In the context of the repeated 80 m sprint test, we considered the maximum speed from the first sprint as the desired maximal sprint for the subsequent nine sprints. Each player underwent approximately 300 s of testing, generating a standardized set of 300 data points per player. Optimization and parameter selection for each player were then conducted based on these data. The second phase of data collection involved actual matches, encompassing four matches per player. This phase yielded a substantial dataset of approximately 20,000 data points per player. Recognizing the inherent stochastic nature of soccer and the challenge of measuring the real intentions or demands of players’ velocities, we made the following assumptions: (a) a player’s desired speed is equivalent to the maximum slope value of increasing velocities (acceleration), and (b) the player’s intention to decelerate corresponds to the minimum slope value of decreasing velocities (deceleration). Differences in player weight can significantly impact the exertion at the same running speed [23]. To account for this, we converted all speed values into kinetic energy (work) using the equation $E=\frac{m\cdot v^{2}}{2}$. An illustration of this conversion during an 80 m sprint test for one player is presented in Figure 1, and a snippet from the match is presented in Figure 2.

### 2.4. Soccer MFM

SNS model defines $M_{0}$ as the cumulative motor unit capacity, expressed as $M_{0}=M_{A}+M_{F}+M_{UC}$, where $M_{A}$ denotes the number of active motor units, $M_{F}$ represents the count of fatigued motor units, and $M_{UC}$ signifies the quantity of inactive and unfatigued motor units. In this context, we will characterize $M_{UC}$ as a player’s currently available motor units $M_{P}$, essentially an energy reservoir encapsulated within the player, defined by Equation (1).

(1) $$M_{P}=M_{0}-M_{A}-M_{F}$$

However, we identified a limitation in the SNS approach concerning the change in active motor units, denoted as $\frac{dM_{A}}{dt}$. The assumption that the brain commands (*B* in LBY’s approach) can be represented by the desired activation profile $M_{AD}$ leads to an inconsistency. In their model, they assert that the brain commands deliver the same activation scheme when unfatigued motor units are available (i.e., $M_{AD}<M_{A}+M_{UC}$), resulting in the change in activation being equal to the change in the desired activation profile, $\frac{dM_{A}}{dt}=\frac{dM_{AD}}{dt}$. The authors, however, acknowledge the model’s limitations, stating that it was created and validated on trials with a constant effort followed by maximum effort. This becomes problematic when examining larger intervals with more dynamic changes.

**Figure 3:**
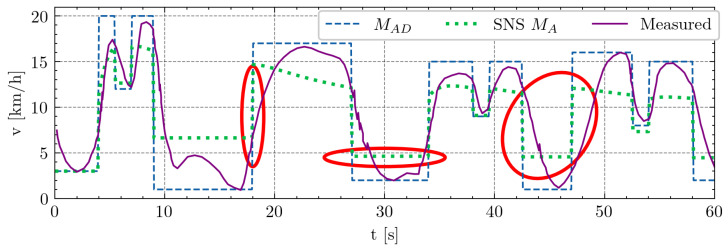
Soccer-specific 1 min drill [20] test. The x-axis denotes time in seconds, while the y-axis represents speed in kilometers per hour (km/h). A green dotted line illustrates the calculated speed derived from the number of activated units, $M_{A}$, with parameters $M_{0}=20$, F=0.05, and R=0.05. The violet solid line represents measured speed values. The rate of change $\frac{dM_{A}}{dt}=\frac{dM_{AD}}{dt}$ proposed by SNS enables unrealistic rates of activation, evidenced by the red ellipse. Such unrealistic rates can pose challenges when numerically calculating the derivative.

Figure 3 illustrates the soccer-specific 1 min drill [20] alongside the estimations generated by SNS model [14]. Initially, the curve closely tracks measured values during the first effort within the initial 10 s. However, subsequent efforts reveal unrealistic activation rate values, as highlighted by the red ellipse. While adjusting parameters *F* and *R* can alter the curve to achieve higher values (*F*) or retain more units (*R*), the inability to modify activation and deactivation rates poses a significant challenge for comprehending soccer dynamics and running contexts. Moreover, challenges arise in computing changes due to issues with derivatives across all scenarios. To address these limitations, we have enhanced the model’s flexibility. A thorough comparison between our proposed solution and the original approach will be presented in the Results section.

Recognizing the influence of a player’s weight on their ability to run at a given speed, we uniquely compute the parameter $M_{0}$ for each player. This calculation is performed using the formula $M_{0}=m\cdot v_{max}^{2}$, effectively representing the player’s maximum energy reservoir. This approach takes into account the player’s mass (*m*) and their maximum speed ($v_{max}$), acknowledging the personalized nature of energy dynamics concerning weight and speed. As demonstrated in SNS, running speed is considered to be the preferred measure of effort as mechanical power is challenging to directly calculate [14]. Speed can be considered proportional to theoretical running “power”, which enables us to relate running speed to force [14]. This rationale justifies representing parameters $M_{0}$, $M_{A}$, $M_{F}$, $M_{P}$, and $M_{AD}$ in terms of force, speed, and energy units. For consistency and simplicity in the paper’s terminology, we maintain the same nomenclature throughout, even though, from a physical standpoint, it would be more accurate to use the symbols for speed (*v*) and energy (*E*).

In the dynamic context of running, the impact of the starting speed being 0 differs significantly from the more common scenario of a middle running distance, such as 10 km/h. As a result, we represent the required change in active motor units as $\Delta M_{AD}=M_{AD}\left(t\right)-M_{A}$, which can be positive (indicating acceleration) or negative (indicating deceleration). This adjustment was necessary to modify Equation (2), describing the alteration in active motor units. This represents the primary enhancement of the SNS approach, enabling its adaptability to soccer dynamics. Here, the parameter $\alpha_{A}$ governs the rate of muscle unit activation during player acceleration, while $\beta_{D}$ denotes the rate of muscle unit deactivation during player deceleration. The previous approach struggled to handle such situations, often resulting in breakdowns and yielding imaginary results. However, the fatigue and recovery Equation (3) remains unchanged, consistent with SNS original approach.

(2) $$\frac{dM_{A}}{dt}=\max\left(0,\Delta M_{AD}\right)\cdot M_{P}\cdot \alpha_{A}+\min\left(0,\Delta M_{AD}\right)\cdot M_{A}\cdot \beta_{D}$$

(3) $$\frac{dM_{F}}{dt}=M_{A}\cdot F-M_{F}\cdot R$$

### 2.5. Procedures

To determine accurate parameters describing each player, we needed to estimate certain unknown factors. The resultant values derived from these parameters must closely align with the real values obtained from GPS sensors. This necessitated the formulation of a model fitting problem to identify parameter values that best describe the player.

The proposed MFM is solved using the Euler method with a time step of Δt=0.1 s, involving four parameters for each player: $\alpha_{A}$, $\beta_{D}$, *F*, and *R*, all of which required estimation. Additionally, we have imposed a constraint $M_{0}-M_{A}-M_{F}\geq 0$ to prevent the accumulation of an unrealistic number of fatigued units $M_{F}$, which are not directly assessed by the model. The boundaries for parameter optimization, both upper and lower, are provided in Equation (4). These boundaries were determined experimentally, drawing upon knowledge from previous research and known parameter ranges.

(4) $$\begin{array}{cc} 1\cdot 10^{-5} & \leq \alpha_{A}\leq 1\cdot 10^{-3},\\ 1\cdot 10^{-5} & \leq \beta_{D}\leq 1\cdot 10^{-2},\\ 1\cdot 10^{-3} & \leq F\leq 1\cdot 10^{-1},\\ 1\cdot 10^{-3} & \leq R\leq 1\cdot 10^{-1}. \end{array}$$

We evaluated the MFM by comparing calculated values with the data acquired from GPS sensors during a repetitive 80 m sprint test. It is essential to note that the MFM equation is solved using the Euler method with a sampling rate ten times higher than the original values. However, only data points aligned with time steps equal to Δt=1 s (following the smoothing of 18 Hz data to 1 s) were utilized to evaluate the fitness of the solution. All optimization variables are consolidated into an optimization vector:

(5) $$\begin{array}{cc} x= & \left(\begin{array}{c} \alpha_{A},\beta_{D},F,R \end{array}\right). \end{array}$$

For each player *i*, the optimization cost function was computed by determining the squared error between MFM values and GPS observations, followed by time averaging, as outlined in Equation (6). The model fitting process was conducted using the stochastic optimization method—PSO. This population-based meta-heuristic algorithm has demonstrated its effectiveness in tackling a wide range of optimization challenges, especially those involving high-dimensional spaces and non-linear landscapes [24]. The optimization process was fine-tuned with a swarm size of 10 and an early stopping criterion triggered after 15 iterations without any improvement in the cost function.

(6) $$\varepsilon_{i}\left(x\right)=\frac{\int_{0}^{t}{\left(E_{i}\left(x,t\right)-E_{measured,i}\left(t\right)\right)}^{2}dt}{t},$$

Optimization procedures were executed repeatedly, with ten optimization runs conducted for each player. Subsequently, the parameters from the most successful run were employed to evaluate the player’s performance across four consecutive matches.

### 2.6. MFM Workflow for Athlete Insights

To gain insights into player performance, two primary data sources are required: the repeated sprint test and match data (illustrated in Figure 1 and Figure 2). The blue dashed line represents $M_{AD}$, which reflects the brain’s intent to perform an activity—in this case, expending energy. This time-series data serve as an input to the MFM.

The MFM also requires fixed parameters ($\alpha_{A}$, $\beta_{D}$, *F*, *R*, and $M_{0}$) as inputs. To estimate an athlete’s individual parameters, $M_{AD}$ from the repeated sprint test is used. An optimization process, leveraging the PSO algorithm, iteratively adjusts the candidate parameters by comparing the model’s output with actual measurements from GPS sensors. Once optimal parameters are identified, they can be applied to match data.

For match analysis, $M_{AD}$ from the game is used as input, while the fixed parameters are taken from the optimization results. The MFM then outputs two time-series: $M_{A}$, representing energy output from active motor units, and $M_{F}$, representing energy consumed by fatigued motor units during the match. These outputs form the basis for calculating the player’s remaining energy ($M_{P}$) as defined in Equation (1).

**Figure 4:**
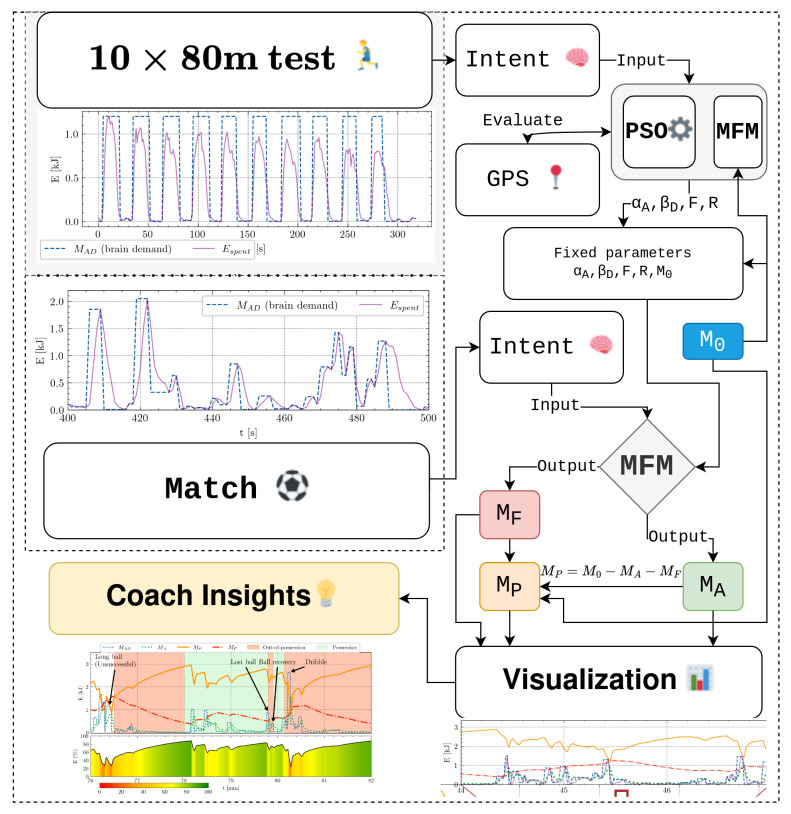
Flowchart illustrating the workflow for analyzing player performance. The process begins with a standardized repeated sprint test, which provides data for the PSO algorithm to estimate the optimal parameters describing the player’s physical profile. These parameters are then applied to match data to calculate time-series outputs for active and fatigued motor unit energy. This enables the visualization of the player’s remaining energy capacity and supports deeper performance insights.

This process enables the visualization of energy expenditure and recovery throughout the match. If combined with tactical event data, it offers deeper insights into player performance and decision-making. Figure 4 provides a visual representation of the described workflow.

## 3. Results

In this section, the SNS approach is compared with our proposed modification, utilizing three distinct tests sourced from the scientific literature. Subsequently, we delve into the performance analysis of the repeated 80 m sprint test, exploring its variability across multiple optimization runs. Finally, we extrapolate the test parameters to assess the real match performance through an examination of four matches.

### 3.1. Comparison with Base Model

This section will present a comparison between the SNS approach and the model enhancement proposed in this manuscript across three distinct tests sourced from various papers. These tests represent maximal effort, as well as soccer-specific drills with shorter and longer durations, highlighting dynamic changes. Specifically, the tests include maximal hand-grip contraction [9], a soccer-specific 1 min drill [20], and a soccer-specific 15 min intermittent treadmill protocol [21]. The data regarding these tests are accessible in the public repository, along with the code required to replicate the plots.

#### 3.1.1. Maximal Hand-Grip Test

**Figure 5:**
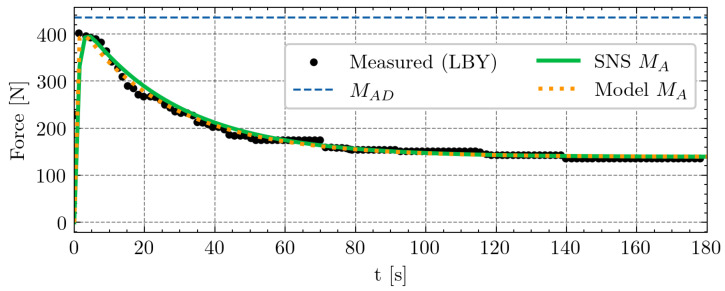
Max hand-grip test conducted by LBY [9] and adopted in the investigation by the SNS approach [14]. The x-axis represents time in seconds, while the y-axis represents force in newtons. The black points on the graph depict measured values, while the solid green line represents the calculated activation $M_{A}$ according to the SNS approach. In contrast, the orange dotted line illustrates the activation $M_{A}$ predicted by our proposed model. For SNS curve, the parameters utilized were $M_{0}=435$, $F=2.45\cdot 10^{-2}$, and $R=1.15\cdot 10^{-2}$, whereas our model incorporated values of $M_{0}=418.55$, $\alpha_{A}=2.10\cdot 10^{-2}$, $\beta_{D}=1.04\cdot 10^{-2}$, $F=2.40\cdot 10^{-2}$, and $R=1.19\cdot 10^{-2}$.

The max hand-grip contraction test, as illustrated in Figure 5, derives its measurements from the LBY study. Our implementation of the SNS model and the model proposed in this paper are both closely aligned to the observed data. The SNS model exhibits an $R^{2}$ score of 0.87, while our model performs slightly better, with an $R^{2}$ score of 0.89, against LBY’s measurements. The optimal parameters outlined in the SNS research [14] were employed to generate the curve for the SNS implementation. Likewise, the parameters for our implementation were determined based on the methodology outlined by the SNS approach, which entails employing a least-squares fit to the hand-grip data to estimate the $M_{0}$, $\alpha_{A}$, $\beta_{D}$, *F*, and *R* parameters. This test serves as a benchmark for our analysis, ensuring that any modifications applied do not compromise performance regarding the established assessments.

#### 3.1.2. Soccer-Specific 1 Min Drill

**Figure 6:**
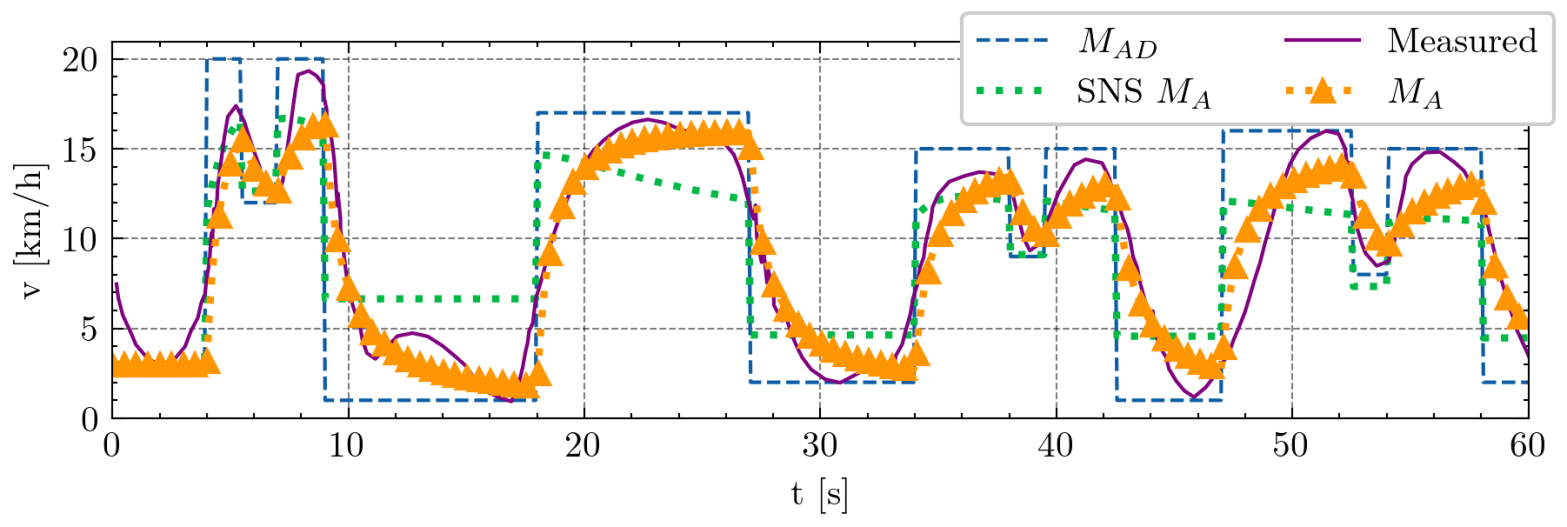
Soccer-specific 1 min drill. The x-axis denotes time in seconds, while the y-axis represents player speed in kilometers per hour (km/h). The demanded $M_{AD}$ values, indicated by the blue dashed line, correspond to the maximum peaks in four exercises within the drill: CID sprints, linear striding, slalom runs, and shuttle runs. Measured values are represented by violet solid lines. The green dotted line illustrates the calculated activation rates according to SNS approach, while the orange dotted line with triangle markers depicts the calculated activation rates for our model. For the SNS approach, the parameters used were $M_{0}=20$, F=0.05, and R=0.05, while, for our model, they were $M_{0}=21.67$, $\alpha_{A}=9.90\cdot 10^{-2}$, $\beta_{D}=8.50\cdot 10^{-2}$, $F=4.23\cdot 10^{-2}$, and $R=5.79\cdot 10^{-2}$.

Figure 6 presents a 1 min segment extracted from a 15 min soccer-specific drill test [20], offering a concise yet dynamic assessment of demanded activation $M_{AD}$. As discussed in Section 2.4, the SNS model encounters challenges with the derivative after the initial peak in dynamic changes, resulting in unrealistic activation rates. This issue is effectively addressed through our model modification and the introduction of parameters $\alpha_{A}$ and $\beta_{D}$, which notably improves the alignment with the observed data. Similar to the approach employed in the max hand-grip test, the model parameters were determined using the least-squares fit methodology.

#### 3.1.3. Soccer-Specific Intermittent Treadmill Protocol

**Figure 7:**
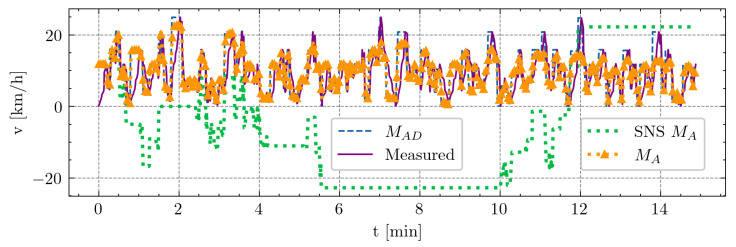
Comparison of models during a soccer-specific 15 min intermittent treadmill protocol. The x-axis represents time in minutes, while the y-axis illustrates a player’s speed in kilometers per hour (km/h). Measured values are depicted by the violet solid line, and the demanded activation $M_{AD}$ is represented by the blue dashed line. Dotted orange lines with triangle markers indicate the calculated activation by the proposed model, $M_{A}$, while green dotted line displays the SNS calculation of $M_{A}$, which leads to unrealistic negative values of speed. For the SNS approach, the parameters used were $M_{0}=27$, F=0.05, and R=0.05, while, for our model, they were $M_{0}=25$, $\alpha_{A}=9.90$, $\beta_{D}=9.96$, $F=9.99\cdot 10^{-2}$, and $R=1.55\cdot 10^{-2}$.

This test served as the final comparison of the models during a longer-duration 15 min protocol. The model parameters were once again determined through least-squares fitting. The objective of this test was to illustrate how the derivative in calculations can lead to unrealistic negative values of speed. Figure 7 displays the comparison of the calculated activation values between the SNS approach and the model proposed in this paper.

Our model demonstrates robustness to longer dynamic changes in demanded activation $M_{AD}$, while the SNS model, due to the derivative in the calculation, is susceptible to unrealistic activation rates, as evidenced by the negative values shown in this plot. This comparison highlights the advantages of our proposed model in handling dynamic changes over extended periods.

### 3.2. Model Performance

**Figure 8:**
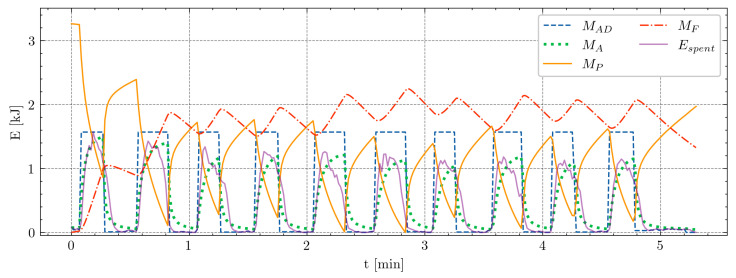
Visual representation of the 80 m sprint test using the MFM for Athlete1, showcasing the best-performing run with parameters $M_{0}=3341$, $\alpha_{A}=0.15\cdot 10^{-3}$, $\beta_{D}=1.17\cdot 10^{-3}$, $F=7.39\cdot 10^{-2}$, and $R=1.91\cdot 10^{-2}$. X-axis corresponds to the time in minutes, and y-axis represents energy in kilojoules. Blue dashed line depicts the demanded energy, green dotted line represents the calculated values, violet solid line illustrates the true values, top orange solid line shows the available energy $M_{P}$ at each moment of the test, and red dotted and dashed line denotes the amount of fatigue $M_{F}$.

Table 1 presents the outcomes of the most successful optimization runs in the 80 m sprint test for the observed players. The $R^{2}$ scores range from 0.62 to 0.80, with all the players showing strong alignment between the model’s predictions and the measured data except for one. However, even for this player, the values closely track the repeated sprinting test measurements, indicating that the model performs reasonably well across all the participants. To provide a visual representation of these outcomes, Figure 8 illustrates Athlete1 and his 80 m sprint test values.

The stability of the optimization process and its outcomes relies on the algorithm’s capacity to yield consistent results, specifically in identifying optimal values. Detailed results of the 10 optimization runs for Athlete1 are presented in Table A1.

### 3.3. Match Applicability

The parameter values from the top-performing run in the 80 m sprint test serve as the foundation for calculating real-match values. Table 2 presents the performance of these fitted parameters across four matches for each player. Interestingly, despite being fitted on the 80 m sprint test, the cost function values (ε) on the match dataset are even smaller for all the players. While the $R^{2}$ values for the matches are slightly lower than those achieved in the 80 m sprint test—except for Athlete3, where they are higher—they remain robust, with the average scores across the four games ranging from 0.70 to 0.72. This level of consistency is notable, especially considering the unpredictable nature of the game.

**Figure 9:**
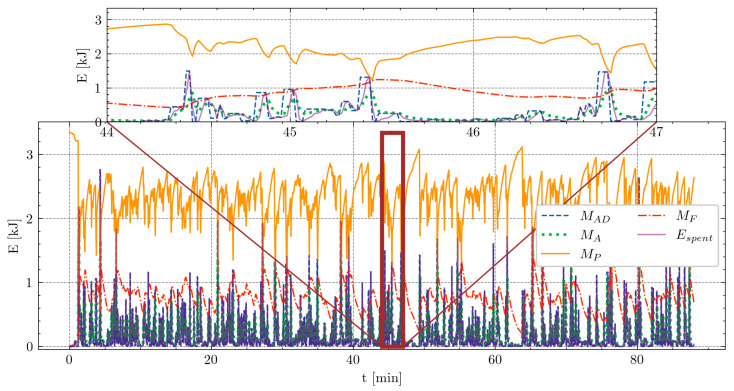
Visual representation of one match using the MFM for Athlete1, employing the best-performing run values with parameters $M_{0}=3341$, $\alpha_{A}=0.15\cdot 10^{-3}$, $\beta_{D}=1.17\cdot 10^{-3}$, $F=7.39\cdot 10^{-2}$, and $R=1.91\cdot 10^{-2}$. X-axis corresponds to time in minutes, and y-axis represents energy in kilojoules. A close-up snippet is presented at the top, focusing on the first match between the 44th and 47th minutes (marked by a brown rectangle in the main plot). The blue dashed line depicts the demanded energy, green dotted line represents the calculated values, violet solid line illustrates the measured values, top orange solid line shows the available energy $M_{P}$ at each moment of the match, and red dotted and dashed line denotes the amount of fatigue $M_{F}$.

The resulting parameters can also be used to visualize players’ energy expenditure throughout a match, offering the possibility to integrate this information with tactical instructions. Figure 9 shows a visualization of one match for Athlete1. Given the complexities of a match and the difficulty in precisely measuring the desired energy output by the player ($M_{AD}$), this visualization is valid when considered relatively. It enables observing fluctuations and recoveries in the player’s energy tank without relying on absolute values.

**Figure 10:**
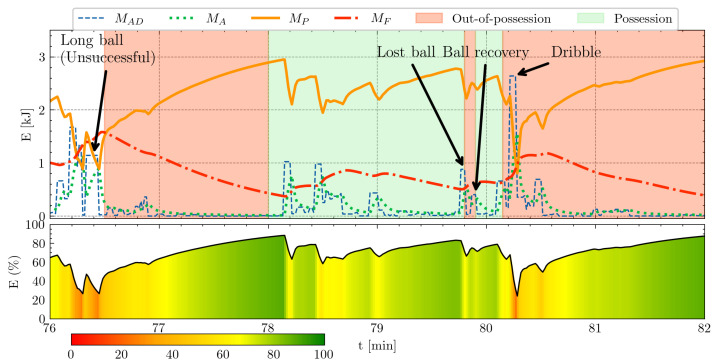
Example use case in match analysis for Athlete1, focusing on the 76th min to 82nd min of a match. The top plot displays absolute energy expenditure, along with key event data (pass, tackle, and dribble), and possession information. The bottom plot represents the player’s remaining energy capacity as a percentage, calculated by dividing $M_{P}$ (remaining absolute energy level) with $M_{0}$ (maximum energy level) and multiplying by 100. By simultaneously examining both event and physical data, coaches can gain a more comprehensive understanding of a player’s performance and energy dynamics during specific moments of the match.

Determining the optimal parameters ($\alpha_{A},\beta_{D},F,R$) is a crucial prerequisite for accurately describing a player’s effort during an 80 m repeated sprint test. Incorporating the fixed $M_{0}$ enables tracking the player’s fatigue and recovery over the course of a session or match. Figure 10 demonstrates how this model can be integrated with tactical data to provide deeper insights. The example includes events such as a long ball, lost ball, recovered ball, and dribble, enabling coaches to connect specific on-pitch scenarios with players’ physical exertion. This connection offers a more comprehensive understanding of player performance and informs tactical and conditioning strategies.

## 4. Discussion

While numerous MFMs have addressed various exercises, our focus lies in tailoring and refining the model for soccer dynamics. Building upon the SNS approach [14], we maintain the model’s simplicity while enhancing its robustness based on the players’ demand or desire to exert themselves in a certain manner. Capturing soccer’s complexity with limited parameters is challenging [25]; therefore, we aim to compress information efficiently while minimizing errors, necessitating certain assumptions.

The primary limitation involves the model’s input—the desired activation profile $M_{AD}$. This profile is based on the initial sprint energy in the 80 m repeated sprint test, peak acceleration, and deceleration values during matches. While assuming that a player aims to maintain the same speed as in the first sprint is reasonable [26], the assumption regarding the desired exertion during matches is debatable. Soccer is chaotic and influenced by many variables. For example, a player’s gradual acceleration might be due to timing considerations rather than fatigue. Moreover, estimating the speed a player intended to achieve is infeasible, except during high-intensity events like sprints, rapid accelerations, or abrupt stops, where the goal is either maximum effort or complete halting. The model requires this intention, or “brain command”, as an input, limiting its adaptability. To accurately assess those parameters defining a player’s physical profile, controlled tests with clear objectives—like the repeated maximal sprint test—are necessary. In such tests, the player’s intent is known, enabling the model to precisely evaluate their effort. While soccer matches are more unpredictable, the model can still track energy fluctuations during the game. However, discrepancies might arise because the model cannot reliably determine the player’s intent during match play. Despite the challenges in estimating $M_{AD}$, the model provides significant insights into match dynamics and lays a foundation for further research.

Another assumption is that a player’s total number of motor units $M_{0}$ or maximum potential $M_{P}$ equates to the kinetic energy, calculated by multiplying the player’s mass with their squared maximal speed [27]. While this is physically valid, it is uncertain if the player’s maximum recorded speed is their absolute maximum or influenced by the opposition [28]. We chose to maintain this simplified approach, focusing on other parameters governing fatigue and recovery dynamics, streamlining the model without complicating optimization.

We validated our model against the LBY and SNS approaches [14] using the max hand-grip test and two soccer-specific drills from the literature: the soccer-specific 1 min drill and the 15 min treadmill protocol. These drills highlighted the limitations of the existing methods while demonstrating the enhanced performance of our model. This validation supported the use of PSO fitting to determine the optimal parameters for athletes performing an 80 m repeated sprint test. The results showed an $R^{2}$ ranging from 0.62 to 0.80 for the best-performing run across all the players. When these optimized parameters were applied to match data, the model showed lower cost function values for all the players, maintaining respectable $R^{2}$ scores from 0.70 to 0.72 across matches.

One potential application of this model is in tactical analysis. Currently, event data in soccer are collected using a combination of computer vision and manual annotation [29]. These data provide detailed information about the events on the pitch, including their location, the players involved, and event-specific details, such as whether a pass was successful, its type (e.g., long pass), and the intended recipient.

Tactical decisions on the pitch are closely linked to players’ physical states [30]. For example, if a player executes a sprint at full exertion and then immediately needs to shoot, their ability to apply proper technique or make optimal decisions may be compromised [30]. This could result in a missed opportunity to either score or pass to a better-positioned teammate. Similarly, when a player fails to track back defensively after losing possession of the ball, it might not necessarily be due to laziness but rather physical fatigue that necessitates recovery time. In a hypothetical scenario following the ‘dribble’ event shown in Figure 10, the player loses possession of the ball and does not immediately track back on defense. By analyzing the energy capacity graph at the bottom of the figure, coaches can identify the underlying cause—specifically that the player was recovering from a prior high-intensity action—and use this understanding to devise strategies aimed at mitigating similar risks in the future.

Our goal is not to propose a definitive methodology for evaluating players’ fitness but to assess fatigue and recovery profiles. Future research could explore drills and exercises that more accurately replicate the physical and tactical demands of match dynamics. Additionally, collecting data from players with diverse physical characteristics and playing positions could provide an opportunity to further validate this method and refine its use in categorizing players into distinct physical profiles. This paper is the first to use the MFM approach in soccer. Despite the sport’s dynamic nature, our model fitting results are promising. We offer full access to the source code and data, facilitating replication and serving as a foundation for future research. By determining player profiles and optimal parameters, we aim to open new avenues where tactical event data can intersect with performance metrics, enabling coaches to make informed strategic adjustments and enhance team performance [31]. For instance, a coach might identify that a specific player struggles to recover from post-attacking pressure after the 60th minute, leading to defensive vulnerabilities, enabling strategic adjustments.

## 5. Conclusions

Our work is centered on tailoring and refining MFMs to suit the distinct dynamics of soccer. We build upon the simplicity of the SNS approach while improving its robustness. Despite the challenges associated with capturing soccer’s complexity using limited parameters, our objective is to effectively compress information while minimizing errors.

The validation process against the SNS approach, employing the maximum hand-grip test, soccer-specific 1 min drill, and 15 min treadmill protocol, underscores that our enhancements preserve the integrity of the original framework while tailoring it to the unique demands of soccer. The optimization with the data from the 80 m sprint test produced consistent results, with $R^{2}$ scores of 0.79, 0.78, 0.62, 0.76, and 0.80 for the athletes. Although the $R^{2}$ score for one athlete was slightly lower, the findings highlight the model’s ability to align closely with observed data and effectively capture player dynamics.

The parameters obtained through the utilization of the PSO method were employed to evaluate performance using four-match data, comprising 20,000 data points per player. The repeated sprint test success extended to match data, where, despite inherently lower $R^{2}$ scores due to the unpredictable nature of the game, the model’s performance remained strong, with the mean $R^{2}$ ranging from 0.70 to 0.72. We provide full access to our source code and data, facilitating result replication and laying the groundwork for future research. Our pioneering application of the MFM approach to soccer yields promising results, potentially empowering coaches to correlate tactical decisions with real-time physical performance for more informed strategies and enhanced team performance. 

## Figures and Tables

**Table 1:** Presentation of the top-performing runs achieved through PSO for the observed athletes. Variable $N_{eval}$ indicates the number of evaluations required to achieve optimization values, where $M_{0}$ denotes the player’s total motor unit capacity, and $\alpha_{A}$, $\beta_{D}$, *F*, and *R* represent the final parameters acquired through optimization. Column ϵ indicates the final cost function value obtained from the 80 m sprinting test, while the last column presents the $R^{2}$ metric associated with the sprinting test results.

Player	$N_{\mathrm{eval}}$	$M_{0}\;\left(\frac{J}{\mathrm{kg}\cdot s}\right)$	$\alpha_{A}\;\left(s^{-1}\right)$	$\beta_{D}\;\left(s^{-1}\right)$	$F\;(s^{-1})$	$R\;(s^{-1})$	$\epsilon \;(\times 10^{6})$	$R^{2}$
Athlete1	850	3341	$1.50\cdot 10^{-4}$	$1.17\cdot 10^{-3}$	$0.74\cdot 10^{-1}$	$1.91\cdot 10^{-2}$	3.30	0.79
Athlete2	770	2503	$2.00\cdot 10^{-4}$	$3.03\cdot 10^{-3}$	$0.60\cdot 10^{-1}$	$1.82\cdot 10^{-2}$	2.23	0.78
Athlete3	1100	2666	$1.60\cdot 10^{-4}$	$4.20\cdot 10^{-3}$	$0.74\cdot 10^{-1}$	$1.95\cdot 10^{-2}$	3.60	0.62
Athlete4	1110	3530	$0.90\cdot 10^{-4}$	$2.54\cdot 10^{-3}$	$0.90\cdot 10^{-1}$	$2.25\cdot 10^{-2}$	4.00	0.76
Athlete5	930	2599	$1.40\cdot 10^{-4}$	$2.22\cdot 10^{-3}$	$0.46\cdot 10^{-1}$	$2.16\cdot 10^{-2}$	3.50	0.80

**Table 2:** Performance of the model using data from four matches. Model parameters were set to the best-performing run in the 80 m test (Table 1). Column ϵ corresponds to the final cost function value (squared error divided by the number of minutes), while the “Minutes” column indicates the total playing time for each player across the four matches. $R^{2}$ scores are provided for each of the four matches, denoted from M1 to M4, along with the mean $R^{2}$ across all observed matches.

Player	Minutes	$\epsilon \;(\times 10^{6})$	$R_{M1}^{2}$	$R_{M2}^{2}$	$R_{M3}^{2}$	$R_{M4}^{2}$	$R_{\mu }^{2}$
Athlete1	250	1.66	0.71	0.72	0.70	0.69	0.70
Athlete2	224	1.01	0.70	0.77	0.67	0.76	0.72
Athlete3	260	1.60	0.74	0.71	0.67	0.72	0.71
Athlete4	222	1.25	0.76	0.70	0.69	0.70	0.71
Athlete5	276	1.06	0.71	0.71	0.74	0.73	0.72

## Data Availability

The authors confirm that the data supporting the findings of this study are publicly available within the article or at the following link: https://github.com/askoki/soccer-fatigue-model (accessed on 4 December 2024).

## Abbreviations

The following abbreviations are used in this manuscript:

SNS	Sih, Ng, and Stuhmiller [14]
LBY	Liu, Brown, and Yue [9]
MFM	muscle fatigue model
GPS	global positioning system
PSO	particle swarm optimization
MVC	maximum voluntary contraction
CID	change in direction
Nomenclature	
$\alpha_{A}$	Rate of muscle unit activation
$\beta_{D}$	Rate of muscle unit deactivation
*F*	Rate at which active motor units fatigue
*R*	Rate at which fatigued motor units recover
$M_{0}$	Total number of motor units (or total force-generating capacity)
$M_{A}$	Number of active and unfatigued motor units
$M_{AD}$	Number of active and unfatigued motor units demanded
$M_{F}$	Number of fatigued motor units
$M_{P}$	Number of currently available motor units
*t*	Time (s)

## Appendix A. Optimization Stability

As depicted in Table A1, the majority of the 10 optimization runs for the observed Athlete1 exhibit a satisfying level of consistency in the resulting parameters. However, one run, specifically run 3, does not reach the global optima and settles in the local optima. Although there are slight variations in the values of *F* and *R* across runs, the overall stability ensures the reproducibility of the results, thereby instilling confidence in the obtained values.

**Table A1:** Results of all ten optimization runs for Athlete1 obtained through PSO. Variable $N_{eval}$ indicates the number of evaluations required to achieve optimization values, where $M_{0}$ denotes the player’s total motor unit capacity, and $\alpha_{A}$, $\beta_{D}$, *F*, and *R* represent the final parameters acquired through optimization. Column ϵ indicates the final cost function value obtained from the 80 m sprinting test, while the last column presents the $R^{2}$ metric associated with the sprinting test results.

Run	$N_{\mathrm{eval}}$	$\alpha_{A}\;\left(s^{-1}\right)$	$\beta_{D}\;\left(s^{-1}\right)$	$F\;(s^{-1})$	$R\;(s^{-1})$	ϵ	$R^{2}$
1	850	$1.50\cdot 10^{-4}$	$1.17\cdot 10^{-3}$	$0.74\cdot 10^{-1}$	$1.91\cdot 10^{-2}$	3,270,712	0.79
2	1560	$1.30\cdot 10^{-4}$	$1.19\cdot 10^{-3}$	$1.00\cdot 10^{-1}$	$2.87\cdot 10^{-2}$	3,294,160	0.79
3	960	$1.60\cdot 10^{-4}$	$1.19\cdot 10^{-3}$	$0.61\cdot 10^{-1}$	$1.47\cdot 10^{-2}$	3,278,023	0.79
4	630	$1.50\cdot 10^{-4}$	$1.17\cdot 10^{-3}$	$0.74\cdot 10^{-1}$	$1.91\cdot 10^{-2}$	3,271,489	0.79
5	810	$1.40\cdot 10^{-4}$	$1.17\cdot 10^{-3}$	$0.92\cdot 10^{-1}$	$2.60\cdot 10^{-2}$	3,284,659	0.79
6	1130	$1.30\cdot 10^{-4}$	$1.19\cdot 10^{-3}$	$1.00\cdot 10^{-1}$	$2.88\cdot 10^{-2}$	3,294,352	0.79
7	1040	$1.30\cdot 10^{-4}$	$1.18\cdot 10^{-3}$	$0.99\cdot 10^{-1}$	$2.83\cdot 10^{-2}$	3,292,478	0.79
8	2270	$1.40\cdot 10^{-4}$	$1.17\cdot 10^{-3}$	$0.86\cdot 10^{-1}$	$2.34\cdot 10^{-2}$	3,278,294	0.79
9	1440	$1.30\cdot 10^{-4}$	$1.16\cdot 10^{-3}$	$0.94\cdot 10^{-1}$	$2.63\cdot 10^{-2}$	3,285,208	0.79
10	660	$1.30\cdot 10^{-4}$	$1.15\cdot 10^{-3}$	$0.96\cdot 10^{-1}$	$2.72\cdot 10^{-2}$	3,288,196	0.79
